# Interpretation of Impact-Echo Testing Data from a Fire-Damaged Reinforced Concrete Slab Using a Discrete Layered Concrete Damage Model

**DOI:** 10.3390/s20205838

**Published:** 2020-10-15

**Authors:** Changkye Lee, Seong-Hoon Kee, Jun Won Kang, Byong-Jeong Choi, Jin Woo Lee

**Affiliations:** 1 University Core Research Center for Disaster-free & Safe Ocean City Construction, Dong-A University, Busan 49315, Korea; changkyelee@dau.ac.kr; 2 Department of Architectural Engineering, Dong-A University, Busan 49315, Korea; 3 Department of Civil Engineering, Hongik University, Seoul 04066, Korea; jwkang@hongik.ac.kr; 4 Department of Architectural Engineering, Kyonggi University, Suwon 16227, Korea; bjchoi@kyonggi.ac.kr; 5 Department of Civil Engineering, Korea Electric Power Corporation Engineering and Construction (KEPCO E&C), Gimcheon-si 39660, Korea; jinwoo@kepco-enc.com

**Keywords:** heat-induced concrete damage, impact-echo testing, standard fire test, discrete layered concrete model

## Abstract

The main objectives of this study are to investigate the spectral responses of a fire-damaged concrete slab using Impact-echo (IE) testing, and to develop a simplified model for interpreting the frequency shift due to heat-induced concrete damage after the fire. For these purposes, a reinforced concrete slab specimen (1000 mm (width) by 5000 mm (length) by 210 mm (thickness)) was fabricated in the laboratory. Heat damage in the concrete slab specimen was induced by exposing the bottom of the specimen to the temperatures corresponding to the standard fire curve described in the ASTM E 119 for 3 h. Impact-echo testing was performed on the bottom surface of the concrete slab specimen before and after inducing the fire damage. It was observed that the spectral responses of the fire-damaged concrete were dominated by several non-propagating waves, which resulted in main peak frequencies around 4500 Hz and 5100 Hz. A discrete layered concrete damage model developed in this study was used to reconstruct the variation of the P-wave velocity with the depth of the fire-damaged concrete. It was demonstrated that the predicted P-wave velocity profile using the simplified model showed a good agreement with the measured values from the five core samples, which measured 100 mm (diameter) by 200 mm (height) cylinders, using ultrasonic pulse velocity (UPV) measurements at eight different depths. In addition, the peak frequencies predicted by the simplified model were consistent with the measured peak frequencies. The experimental results in this study demonstrated that IE testing is effective for evaluating the post-fire damage of reinforced concrete slabs. Particularly, the simplified model in this study can be effective for better interpreting the spectral responses of fire-damaged concrete slabs by IE testing.

## 1. Introduction

Fire is one of the most critical types of disaster for buildings and civil infrastructure systems. Concrete is regarded as one of the most resistant to heat and fire among the building and construction materials [1]. However, thermal damage caused by the heating and cooling of concrete could substantially reduce its mechanical properties (e.g., compressive strength and elastic modulus) [2,3,4]. Therefore, it is important to evaluate the condition of structural materials and to check the structural integrity of structures after fire, and if necessary, to decide appropriate rehabilitation schemes. 

**Figure 1:**
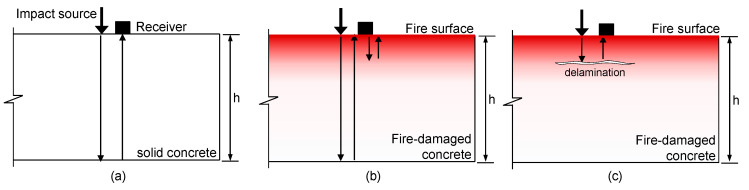
Illustration of the source-and-receiver configuration in IE testing: (**a**) on solid concrete, (**b**) on fire-damaged concrete without delamination defect and (**c**) on fire-damaged concrete with delamination defect.

Impact-echo (IE) is a widely used nondestructive testing (NDT) method that has been successfully used to evaluate the concrete in structures [5,6,7]. It is an elastic wave-based method that examines the transient vibration response of a plate-like structure subjected to a mechanical impact. Figure 1a illustrates the typical source-and-receiver configuration of IE testing performed on sound concrete. There are three kinds of mechanical waves simultaneously propagating in the plate due to the mechanical impact: two kinds of body waves (P-waves and S-waves) and surface-guided waves (e.g., Lamb and Rayleigh surface waves). Theoretically, an infinite set of non-propagating wave modes (or vibration resonance modes) of the plate is set up by the multiple-reflected and mode-converted body waves guided by the two free surfaces of the plate. In practice, a vibration sensor (e.g., a displacement sensor, accelerometer, microphone, etc.) is located close to the impact source to measure the dynamic response of the plate [5] (see Figure 1). The Fourier transform (amplitude spectrum) of the measured transient time signal will show maxima (peaks) at certain frequencies that are dominated by non-propagating waves (or resonance modes). The interpretation of non-propagating waves is an important process in the IE data analysis. In practice, the fundamental thickness stretch mode normally dominates the spectral response of a plate if there are no near-surface defects, such as cracking, debonding and honeycombs, in the concrete. It is known that the fundamental thickness stretch mode is related to the zero-group-velocity frequency of the first symmetric (S_1_) Lamb mode (or S1 ZGV Lamb mode frequency, *f_IE_*) [8]. There is a simple equation that can relate the thickness of a plate, *h,* and the P-wave velocity of concrete, *C_p_*, as follows:

(1) $$f_{IE}=\beta C_{P}/2h$$

where *β* is the correction factor [6] that depends on the Poisson’s ratio of the concrete [8], and is in the range of 0.945 to 0.957 for concrete. 

The NDT methods using Impact-echo testing have been effectively used to evaluate fire-damaged concrete in the laboratory and in the field. It has been shown that the P-wave velocity of concrete is a good indicator for evaluating the chemical and mechanical changes in fire-damaged concrete [9,10,11]. Some researchers investigated the variations in the resonance frequencies of regularly shaped concrete specimens (cylinders or prisms) with various maximum exposure temperatures [12,13,14,15,16,17,18]. Recently, the authors investigated the variations in the dynamic mechanical properties of concrete (elastic modulus and Poisson’s ratio) for nuclear power plants in Korea using ultrasonic pulse velocity measurements and resonance frequency tests [19]. The specimens in that research were made with the same mixing proportions used for the fabrication of the concrete slab specimen in this study. In the previous study, the dynamic properties were correlated with static mechanical properties (i.e., static elastic modulus and compressive strength) [19]. Impact-echo testing was also used successfully to evaluate the post-fire condition of the concrete structures by measuring the resonance frequencies of circular, thin-disk shaped concrete samples obtained from the core samples [20]. In addition, some researchers performed condition assessments of fire-damaged concrete samples from reinforced concrete elements using the same conventional IE testing setup as that shown in Figure 1a [21,22,23,24,25]. It has been demonstrated in the studies that the spectral responses of fire-damaged concrete manifested as shifts of the peak frequencies to frequencies lower than those measured on sound concrete. The frequency shift in IE testing can be a useful parameter to evaluate the post-fire damage of concrete elements in buildings and civil infrastructure [23]. 

**Figure 2:**
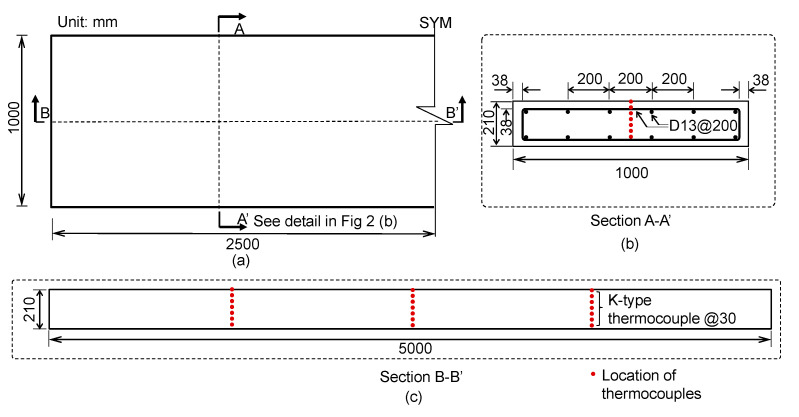
Design of the reinforced concrete slab specimen in this study: (**a**) plan view, (**b**) cross section (A-A’) and (**c**) cross section (B-B’) of the reinforced concrete slab specimen.

**Figure 3:**
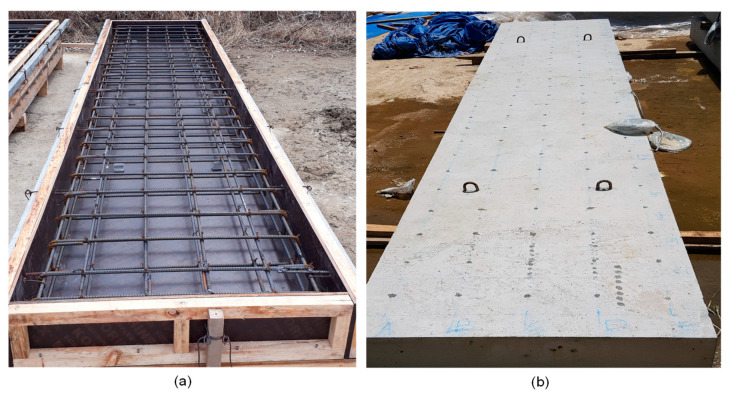
Photo of the reinforced concrete slab specimen in this study: (**a**) reinforcing steel cage in a wooden form before casting concrete and (**b**) hardened concrete slab specimen.

However, it is still difficult to accurately evaluate the degree of fire damage in concrete by measuring the frequency shift using IE testing. The shift of peak frequencies measured for the fire-damaged concrete by IE testing can be explained by three possible reasons: (1) reduced stress wave velocities due to the degradation of the material properties of concrete after being exposed to high heat (e.g., elastic modulus of concrete) (see Figure 1b), (2) the appearance of new multiple reflection waves due to the abrupt change of the P-wave velocity gradient (or high acoustic impedance) near the fire-exposed surface (see Figure 1b), and (3) the presence of a crack parallel to the concrete surface (or horizontal crack) caused by internal thermal stress (see Figure 1c). The frequency shift is dependent on the temperature profile of concrete, the temperature-dependent material properties of concrete, and the thickness of the concrete slab. However, it is still difficult to find, in the literature, a simple formula (or analytical model) describing the variation in IE frequencies measured for fire-damaged concrete using IE testing.

The main objectives of this study are to investigate the spectral responses of a fire-damaged concrete slab using IE testing and to develop a simplified model for the better interpretation of the frequency shift caused by heat-induced concrete damage. For these purposes, this study includes three main tasks: (1) the fabrication of a reinforced concrete slab specimen (1000 mm (width) by 5000 mm (length) by 210 mm (thickness)) and the preparation of fire-damaged concrete by exposing the bottom surface of the specimen to the standard fire test in ASTM E 119 for 3 h; (2) IE testing on the sound and fire-damaged concrete slabs; and (3) the development of a simplified fire-damaged concrete model to interpret the spectral response of post-fire concrete damage. As will be discussed, the simplified model in this study is effective to better understand the spectral responses of fire-damaged concrete slabs by IE testing. 

## 2. Experiments

### 2.1. Preparation of a Concrete Slab Specimen

A concrete slab specimen was fabricated as part of a research project investigating the structural behavior of fire-damaged reinforced concrete elements in nuclear auxiliary buildings in Korea. The concrete slab specimen was designed to have dimensions of 1000 mm (width) by 5000 mm (length) by 210 mm (thickness), which is a 40% scaled prototype of the reinforced concrete slab in used nuclear reactor auxiliary buildings in Korea (see Figure 2). The concrete slab specimen has D13 bars, with a yield strength of 400 MPa (SD400) [26] at the top and at the bottom of the slab in both directions (see Figure 3a). The clear cover to the longitudinal rebars was 38 mm. The concrete slab specimen was fabricated using concrete with the design compressive strength of 35 MPa (5000 psi), which is used for the design and construction of reinforced concrete slabs in nuclear auxiliary buildings in Korea. The concrete was directly sourced from a batch plant located in an actual nuclear power plant construction site in Korea. The concrete was made with type I Portland cement, siliceous coarse aggregates with a nominal maximum aggregate size of 25 mm (1 inch), and a water-to-binder ratio of 0.4. The unit masses of fine and coarse aggregates are 570.6 kg/m^3^ and 737.54 kg/m^3^, respectively, with a sand/aggregates volume ratio of 43.5%. The specific concrete mix proportion is not provided in this study for confidentiality reasons. The average 28-day compressive strength measured from the five concrete cylinders (100 mm diameter by 200 mm height) in the laboratory was 41.2 MPa.

The concrete slab specimen was cured via the wet-curing process by placing a blanket over the slab surface for a week. The curing blanket was composed of water-proofing fabric that was wetted to provide consistent moisture to the slab surface and a less permeable layer that slows the rate of evaporation. The concrete slab specimen was demolded at seven days after casting the concrete, and then cured in the air until the fire test. The curing condition of the concrete cylinders was consistent with the concrete slab specimen. The concrete cylinders were cured in a water tank for a week after casting the concrete, and then demolded. After being demolded, the concrete cylinders were cured in the air next to the concrete slab specimen so that it was exposed to temperature and humidity conditions consistent with those of the concrete slab specimen. 

### 2.2. Standard Fire Test of the Concrete Slab Specimen

**Figure 4:**
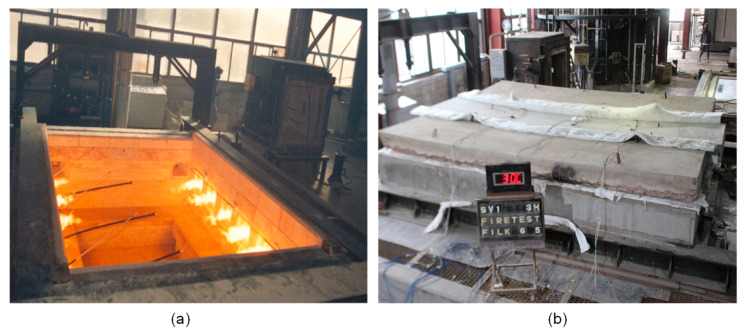
A furnace for horizontal building members: (**a**) a chamber in the furnace with eight natural gas burners and three K-type thermocouples for monitoring temperature during fire testing, and (**b**) the concrete slab specimen under standard fire testing.

**Figure 5:**
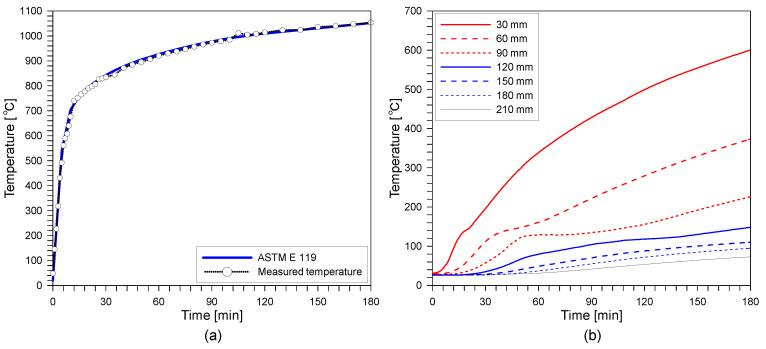
Temperature during ASTM E 119 standard fire: (**a**) ASTM E 119 standard fire curve and measured temperature in the furnace chamber, and (**b**) temperature within concrete slabs measured by K-type thermocouples embedded in the concrete slab specimen.

A standard fire experiment was performed for the reinforced concrete slab specimen to induce fire damage. A standard fire resistance test was carried out using a furnace for horizontal building materials at the Fire Insurers Laboratories of Korea (FILK). The bottom side of the concrete slab specimen was exposed to a standard fire according to ASTM E 119 for 3 h [27]. The dimension of the chamber was 3 m (width) × 4 m (depth) × 1.5 m (height) (Figure 4). In the chamber of the furnace, eight natural gas burners were located to provide thermal energy. The air temperature of the furnace was measured by nine K-type thermocouples (chromel and alumel alloy) covered by a protective tube with an outer diameter of 6.4 mm in accordance with KS C 1602 [28]. The furnace temperature was programmed to follow the standard fire curve specified in ASTM E 119. Figure 5a shows the variation of temperature in the furnace chamber during the fire test and the standard fire curve described in ASTM E 119. In addition, the temperature within concrete slab was monitored by 21 type-K thermocouples (chromel–alumel alloy) with a diameter of 1.3 mm in accordance with KS C 1602 [28]. Note that the K-type thermocouple used in this study has a high accuracy, with an error of ±2.2 °C in a wide temperature range (0 °C to 1260 °C). The temperatures were measured at the three locations indicated in Figure 2b,c. At each location, seven thermocouples were placed at different depths (30, 60, 90, 120, 150, 180 and 210 mm) from the bottom of the concrete slab specimen (Figure 2c). Figure 5b exhibits the temperature within the concrete slabs during the ASTM E 119 fire test for 3 h.

### 2.3. Test Setup and Procedure for Impact-Echo Testing 

**Figure 6:**
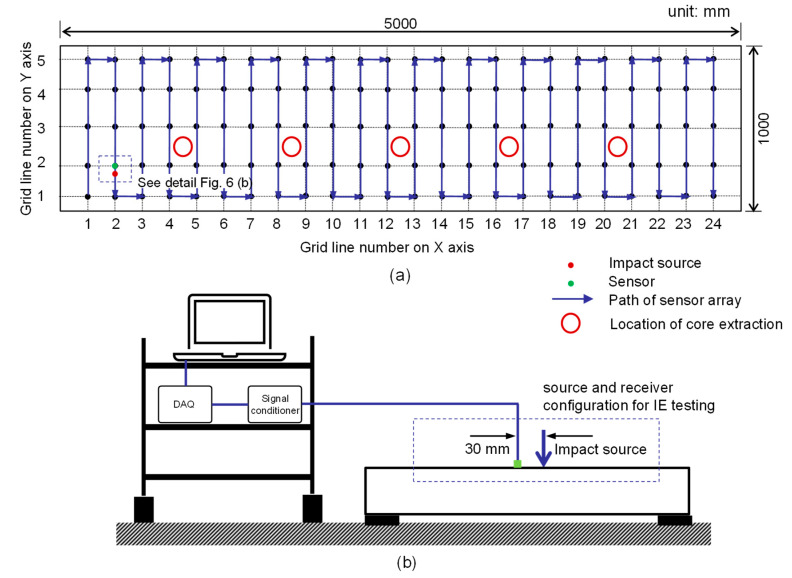
Test setup and procedure of IE testing on the concrete slab specimen: (**a**) test grid and path of sensor array during IE testing and (**b**) test setup of IE testing.

Figure 6 shows the test setup and procedure for IE testing on the surface of the concrete slab specimen. The IE test was performed with prototype equipment developed by a research team at Dong-A University. The prototype equipment is composed of an accelerometer, a signal conditioner, a data acquisition system and a laptop computer. The accelerometer (Model: PCB 352C33) used in this study has a small diameter of 12 mm, a high sensitivity (10.2 mV/(m/s^2^)), a broad bandwidth (0.5 Hz to 10 kHz), and a high resonance frequency (≥50 kHz) [29]. Mechanical impact was generated with a steel ball of 12 mm diameter, which was effective for generating the broad band frequency range of interest for IE testing in this study (1 kHz to 15 kHz). The accelerometer was used to measure the transient time signal representing the dynamic response of concrete, which was simultaneously amplified by a signal conditioner (480E09). The amplified signal was digitized using a high-speed oscilloscope (NI-USB 5132) at a sampling frequency of 1 MHz for a duration of 5 ms. The digitized signals were stored in a laptop computer for data reduction and interpretation. 

**Figure 7:**
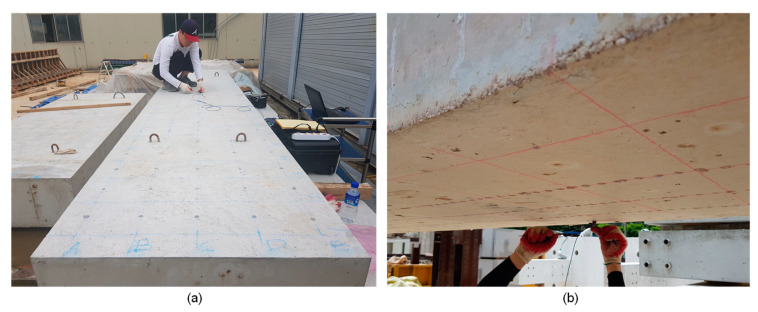
Pictures of IE testing on the test-specimens: (**a**) solid concrete and (**b**) fire-damaged concrete.

IE testing was performed on the bottom surface of the concrete slab specimen before and after the standard fire test. Figure 6a shows the test points for IE testing on the surface of the concrete slab specimen. For the solid concrete slab specimen, IE testing started at (x, y) = (1, 1) by placing an accelerometer at the test point. An incident stress wave was generated by hitting an impact source at about 30 mm from the test point (or sensor location). In this study, the normalized sensor-to-receiver distance (*r*/*h*, *r* is the sensor-to-receiver distance and *h* is the thickness of a slab) was about 0.142, which generates sufficient excitability of the thickness stretch mode for the IE test and analysis in this study [30]. The accelerometer was then moved to the next test point along the transverse direction, i.e., (x, y) = (1, 2). Then, the test was repeated for all the test points along the grid line at x = 1 and along the remaining test lines in the longitudinal direction. Consequently, the IE data scans include a total of 120 test points on the surface of the sound concrete slab specimen. On the fire-damaged concrete surface, IE testing was performed using the same procedure as that for the sound concrete. However, the test area was limited to the region between test grid lines x = 3 and x = 22, where the concrete surface was exposed to fire. Note that only 4 m of the concrete slab specimen in the longitudinal direction was exposed to the standard fire due to the limitation of the size of the furnace chamber. Therefore, the data scans represent a total of 100 test points on the surface of the damaged concrete slab specimen. In addition, the IE testing was performed at five locations, (x, y) = ((4, 3), (8, 3), (12, 3), (16, 3), (20, 3)), at the top (or the opposite surface to the fire-exposed surface) of the fire-damaged concrete slab specimen, to compare the spectral responses from the exposed and the opposite surfaces of the concrete slab. Figure 7a and b show pictures from the IE testing on the solid concrete and fire-damaged concrete slab specimens, respectively. 

**Figure 8:**
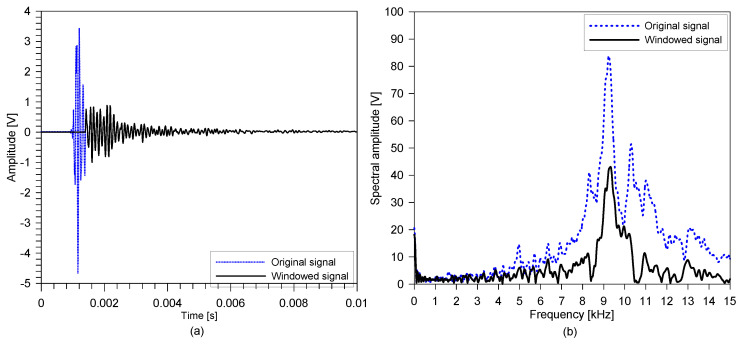
Typical signals from the IE testing of the sound concrete slab specimen: (**a**) typical time signal and (**b**) spectral amplitude signal.

Figure 8 shows the typical time and frequency signals, measured at the test point, (x, y) = (12, 3), on the sound concrete slab specimen. In this study, windowed signals were obtained by applying Hanning windows, with four durations of period, at the minima of the first dominant time wave signals. As such, the surface wave component was extracted from the original time signal, and is presented as a blue dashed line in Figure 8a. In this study, non-propagating waves (resonance modes), presented as a black line in Figure 8a, were simply extracted by subtracting the windowed signal from the original signal. Figure 8b shows the spectral amplitude of the typical time signals shown in Figure 8a. The spectral amplitude of the non-propagating waves exhibits a unique clear peak frequency. As will be discussed in Section 3.1, this peak frequency corresponds to the thickness stretch mode (or S1 ZGV Lamb mode) in IE testing. 

### 2.4. Ultrasonic Pulse Velocity Measurements of Core Samples 

**Figure 9:**
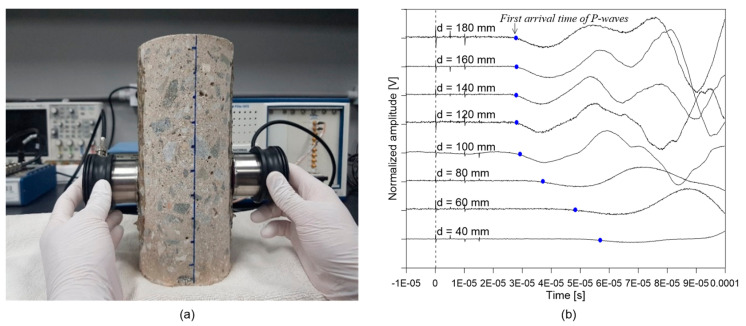
Test setup and typical signals from ultrasonic pulse velocity (UPV) measurements: (**a**) a picture of UPV measurements of a core sample with various depths and (**b**) typical time signals measured at the eight different depths (40, 60, 80, 100, 120, 140, 160 and 180 mm from the fire surface) by 50 kHz P-wave transducers.

Five core samples, 100 mm (diameter) by 200 mm (height) concrete cylinders, were extracted from the fire-damaged concrete slab specimen after IE testing. Figure 6a shows the locations of the core extractions. Five core samples were moved to the laboratory to investigate the variations in the P-wave velocity of concrete with depth of concrete for the fire-damaged concrete slab specimen. The P-wave velocity of concrete was measured at eight different depths (40, 60, 80, 100, 120, 140, 160 and 180 mm) by ultrasonic pulse velocity (UPV) measurements (see Figure 9a). 

The change of the velocity of concrete with depth was measured by a pair of 50 kHz P-wave velocity transducers (Olympus X1021 manufactured by Olympus Corporation, Waltham, MA, USA) placed on the side of a concrete cylinder (see Figure 9a). A pulse-receiver (Panametric 5077 PR manufactured by Olympus Corporation, Waltham, MA, USA) was used to activate a source transducer with a 200 V square pulse, which generated incident ultrasonic pulse waves propagating through the concrete. The ultrasonic pulse waves were measured by the receiving transducer placed on the opposite side of the concrete. A high-speed digital oscilloscope was used to digitize the received signal at a sampling rate of 10 MHz and a total duration of 0.01 s. In this study, the P-wave velocity of concrete at the eight different depths was measured by moving the two sensors to the depth of interest (see Figure 9a). Figure 9b shows the typical time signals measured at the eight different depths of a core sample. It was observed that the first arrival times of the P-waves, marked by the blue dots in Figure 9b, were delayed as the depth of concrete was close to the fire surface. The velocity of an ultrasonic wave was calculated by dividing the wave path L (=100 mm) by the travel time (t =$\;t_{m}-t_{r}$) as follows:

(2) $$C_{P}=\frac{L}{t_{m}-t_{r}}$$

where t_m_ is the time of arrival of the first wave, and t_r_ is the delay time which was measured during the calibration of the probes. The delay time was measured when the two transducers were placed against each other and the time for the first arrival wave was recorded. The arrival of the transient stress waves through cylinders was determined from the measured ultrasonic signals using the modified threshold method [31]. In this method, an approximate arrival time was first obtained using the conventional threshold method in the literature [32]. Next, an accurate arrival time was calculated by fitting a line to the signal data. The intersection of the fitting line and the calculated zero-signal level defines the P-wave travel time.

## 3. Results and Discussion

### 3.1. Typical Signals from Impact-Echo Testing 

**Figure 10:**
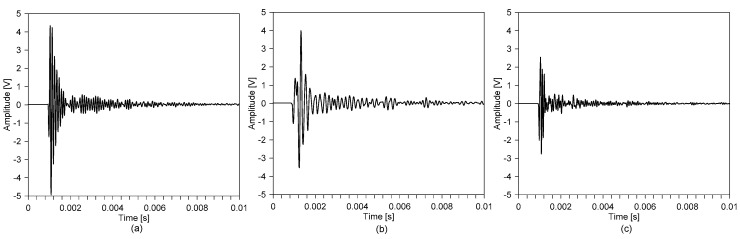
Typical time signals measured on the reinforced concrete slab specimen before and after the standard fire testing: (**a**) on the top surface of sound concrete, and on the bottom (fire-surface) (**b**) and top (**c**) surfaces of the fire-damaged concrete, respectively.

Figure 10 shows typical time signals measured by IE testing on the surface of reinforced concrete slab specimens before and after the 3-h standard fire testing. Consistent with the observations made by previous researchers [15,16,19,22,25], the time signals measured on the concrete surface exposed to fire (Figure 10b) were dominated by waves with longer wavelengths than those measured on the sound concrete (Figure 10a). Furthermore, some differences were also observed in the time signal measured on the surface opposite the fire exposure side, compared to that measured on the sound concrete.

**Figure 11:**
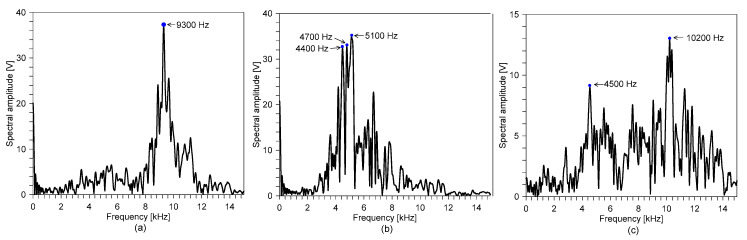
Typical spectral amplitude measured on the reinforced concrete slab specimen before and after the standard fire testing corresponding to the windowed time signals shown in Figure 10a–c.

Figure 11a, b and c show the spectral amplitudes corresponding to the windowed time signals measured on the surface of the sound concrete and the two surfaces of the fire-damaged concrete shown in Figure 10a, b and c, respectively. In the figures, dominant peak frequencies are marked as blue dots. The spectral amplitude measured on the sound concrete was characterized with a single clear peak frequency of 9300 Hz. The dynamic response of the sound concrete slab is simply described by multiple reflections of P-waves between the top and bottom surfaces. Based on the P-wave velocity, C_p_, of 4100 m/s, and the Poisson’s ratio, ν, of 0.2 (i.e., β = 0.9532), the estimated thickness stretch mode frequency on the sound concrete slab is about 9305 Hz, which is in good agreement with the measured peak frequency on the sound concrete. Therefore, the measured peak frequency corresponds to the S1 ZGV Lamb mode frequency.

On the fire-exposed concrete surface, the spectral response was dominated by several peaks, with dominant peak frequencies of 4400 Hz, 4700 Hz and 5100 Hz, which are about 50% of those measured on the sound concrete (see Figure 11 b). The frequency shift in the low-frequency direction can be explained by the reduced P-wave velocity of fire-damaged concrete. The P-wave velocities estimated by Equation (1), based on the reduced peak frequencies measured by IE testing, are 1925 m/s, 1968 m/s and 2231 m, respectively, each of which are about 45% to 55% of the P-wave velocity of sound concrete. The constant P-wave velocity values obtained from Equation (1) are regarded as the average (or apparent) velocity of fire-damaged concrete. Consistent with observations from previous studies, the apparent velocity could be a good indicator for evaluating the average quality of fire-damaged concrete. However, it should be noted that the apparent velocity is not sufficient to accurately evaluate the degree of post-fire damage of concrete, which is characterized by variations in the material properties of concrete with depth. 

In contrast, the spectral response of the surface opposite the fire exposure side is dominated by peak frequencies shifted to the higher frequency range. The temperature profile indicates that the fire-induced concrete damage is localized to the near side of the fire-exposed concrete surface. Furthermore, the temperature near the concrete surface opposite the fire-exposure side is close to that of sound concrete. Therefore, a dynamic response of the concrete surface opposite the fire exposure side is simply described by multiple reflected P-waves between the top concrete surface and an acoustic reflector, due to the high acoustic impedance near the fire-exposed surface. An approximate depth to the acoustic reflector was estimated by Equation (1) based on the measured peak frequency value (10,200 Hz) and the constant mechanical properties of sound concrete (i.e., C_p_, of 4100 m/s and Poisson’s ratio, ν, of 0.2 (i.e., β = 0.9532)). The resulting approximate depth to the acoustic reflector is about 190 mm from the top surface of the concrete (or 20 mm from the fire surface). The estimated fire depth (~20 mm) corresponds to the depth with a maximum temperature of 730 °C, which is higher than the critical temperature of concrete of 600 °C. It can be seen that the approximate approach based on the constant P-wave velocity of sound concrete (~4100 m/s) underestimates the depth of the severely damaged concrete after fire. Note that this approximate approach does not reflect the variation in concrete properties for fire-damaged concrete, as shown in Figure 9b. More detailed analysis, considering the variation in the P-wave velocity for concrete, will be proposed and used to model the dynamic response of fire-damaged concrete in Section 3.3 and Section 3.4. 

### 3.2. Spatial Distribution Peak Frequecines

**Figure 12:**
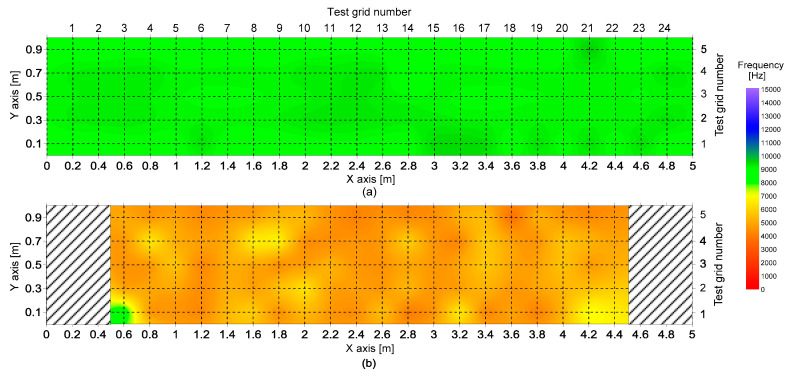
Frequency maps representing the spatial variations in peak frequencies on (**a**) the surface of the sound concrete slab and (**b**) the fire-exposed surface of a concrete slab.

Figure 12a,b show frequency maps representing the spatial variations of peak frequencies on the surface of the sound concrete slab and the fire-exposed surface of the concrete slab, respectively. In the frequency map, frequency value is presented as a color map. Frequencies in the range of 8000 Hz to 10,000 Hz are shown as green, representing sound concrete. Higher frequency values are shown as blue, and lower frequency values are shown as red. The frequency map is effective for visualizing the fire-damaged concrete.

**Figure 13:**
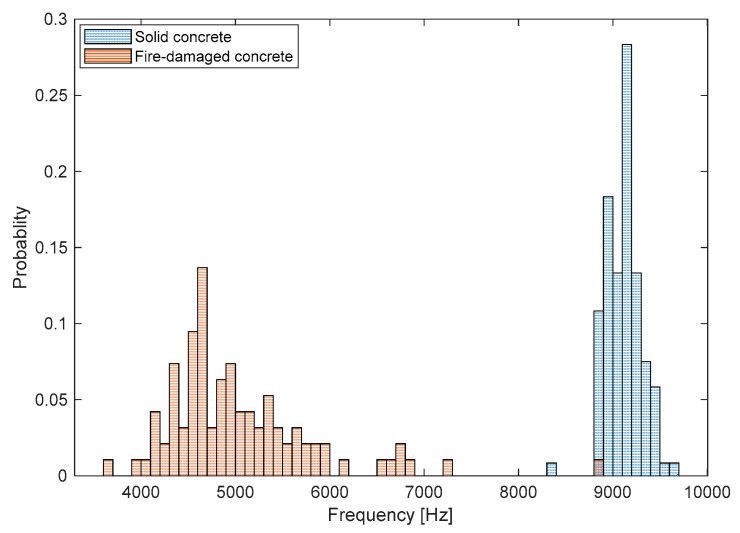
Histograms representing the distribution of peak frequency values measured on the bottom surfaces of solid and fire-damaged concrete slab specimens.

Figure 13 shows histograms representing the distribution of the peak frequency values measured on the bottom surfaces of solid and fire-damaged concrete slab specimens. According to the Kolmogorov–Smirnov (K-S) test, the peak frequency data for the solid and fire-damaged concrete follow a normal distribution [33]. Table 1 summarizes the results of the K-S test with the mean, standard deviation and coefficient of variation (COV) of the surface wave velocity values in the two data groups. After exposure to the 3-h fire test, the average peak frequency value reduced from 9117 Hz to 5034 Hz and the standard deviation increased from 189 Hz to 792 Hz; consequently, the coefficient of variation increased from 2.08% to 15.74%. It was observed that there is a significant difference between the means of Group 1 and 2 at the 1% significance level according to the t-test. 

### 3.3. P-Wave Velocity within the Fire-Damaged Concrete Slab

**Figure 14:**
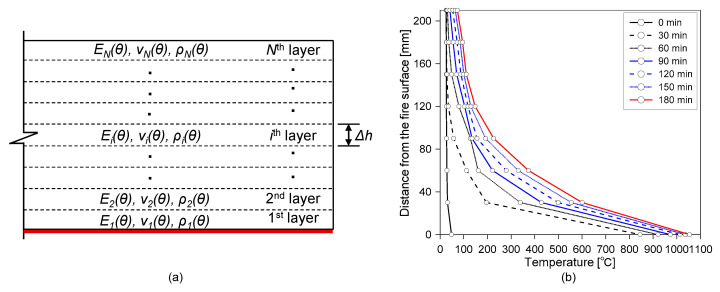
Summary of the estimated P-wave velocity profile in fire-damaged concrete slabs: (**a**) illustration of a discrete layer model for a fire-damaged concrete slab, and (**b**) measured temperature within the fire-damaged concrete during the fire test in this study.

In this study, a discrete layered concrete damage model is developed to predict the properties of fire-damaged concrete in more detail. A discrete layered fire-damaged concrete model is composed of *N* discrete layers parallel to the surface of the concrete (see Figure 14a) to simulate the variations in the concrete’s material properties due to variations in temperature within the concrete. Each discrete layer in the model is assumed to be isotropic, homogenous and elastic, and thus is modeled by the three temperature-dependent material properties that determine the dynamic response of concrete during IE testing (i.e., dynamic elastic modulus ($E_{d\;}$), dynamic Poisson’s ratio ($\nu_{d\;}$) and mass density (ρ)). Consequently, the P-wave velocity of concrete in the *i*^th^ layer ($C_{p,i})$ can be expressed as follows:

(3) $$C_{p,i}=\sqrt{\frac{E_{d\;}\left(\theta \right)\left(1-\nu_{d}\left(\theta \right)\right)}{\rho \left(\theta \right)\left(1+\nu_{d}\left(\theta \right)\right)\left(1-2\nu_{d}\left(\theta \right)\right)}}.$$

where θ is the temperature of the concrete. In this study, approximate equations relating the three material properties of concrete ($E_{d\;}$, $\nu_{d\;}$, and ρ) and the temperature of the concrete are established based on the experimental data measured from concrete cylinders (100 mm diameter by 200 mm height) made with the same concrete mix proportions as those used for the fabrication of the concrete slab specimen in this study. The approximate equations are summarized in the Appendix A of this article, and more detailed information on the experimental study used to obtain the approximate equations is given in the previous study [19]. 

**Figure 15:**
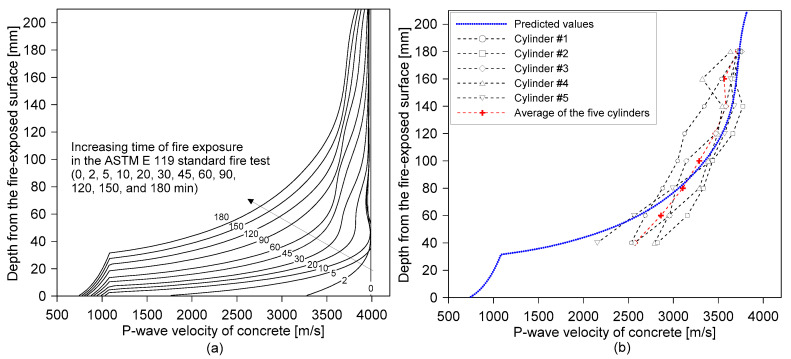
P-wave velocity of concrete with depth: (**a**) predicted P-wave velocity profile of fire-damaged concrete with increasing time of fire exposure after the ASTM E 119 standard fire test, and (**b**) comparison of the P-wave velocity profiles of concrete, estimated by the discrete layered model and measured by ultrasonic pulse wave velocity measurement of the cored cylinders from the fire-damaged concrete specimen after the 3-hour exposure to standard fire testing.

Figure 15 shows the distribution of the predicted P-wave velocity for concrete within the fire-damaged concrete slab specimen after being exposed to ASTM E 119 standard fires for different exposure times (0, 2, 5, 10, 20, 30, 45, 60, 90, 120, 150 and 180 min). The P-wave velocity profiles were estimated by using the discrete layered concrete model based on the temperature profile measured by thermocouples embedded in the concrete slab specimen (see Figure 5b and Figure 14b) and the temperature-dependent material properties described in Appendix A of this article. There are two important observations from the estimated P-wave velocity profile, as follows: (1) the P-wave velocity of fire-damaged concrete decreases at depths closer to the fire exposure surface, which could result in a frequency shift in the lower frequency direction, and (2) fire-induced concrete damage is particularly localized near the fire-exposed surface, which could result in the high impedance mismatch at a boundary layer between the severely- and mildly-damaged concrete layer. 

Figure 15b compares the P-wave velocity profiles of concrete estimated by the discrete layered model and the values measured by UPV after the 3-hour exposure to the standard fire testing. The predicted P-wave velocity at the fire surface is about 1000 m/s, only 25% of that of the sound concrete, and this rapidly increases with distance from the fire surface until the depth of about 50 mm. The predicted P-wave velocity on the opposite side of the exposed surface (d = 210 mm) is 3700 m/s, about 90% of that of the sound concrete. The measured P-wave distributions were from the five core samples extracted from the fire-damaged concrete by UPV measurements. Overall, there is a good agreement between the predicted and measured P-wave velocity of concrete. This result demonstrates that the discrete layered model is effective for estimating the P-wave velocity profile of fire-damaged concrete. However, some differences can be observed between the predicted and measured P-wave velocities of concrete, especially at the location near the surface exposed to fire. These errors could be attributed to several factors, as follows: (1) the heterogeneity of fire-damaged concrete due to enhanced porosity and microcracking, (2) the simplicity of the proposed model, and (3) systematic errors caused by experimental uncertainties (e.g., the imperfect coupling conditions of ultrasonic transducers). However, it is noteworthy that there was a time difference of about four weeks between the fire test and the UPV measurements for the cored samples. The time delay was mainly caused by the preparation and application of non-destructive tests for the fire-damaged concrete, the core extractions, and the delivery of the core samples to the laboratory for UPV measurement. Furthermore, cooling water was supplied during the extracting of core samples from the slab specimen. The surface of the extracted core was wiped with a dry paper tissue to remove the extra water on the surface. Then, the core samples were stored in a resealable plastic bag with a zipper until the UPV measurements. Therefore, the authors cannot exclude the possibility of changes in chemical properties of the fire-damaged concrete due to post-fire curing processes [34,35].

### 3.4. Interpretation of IE Frequency Shift Using the Discrete Layered Concrete Model

**Figure 16:**
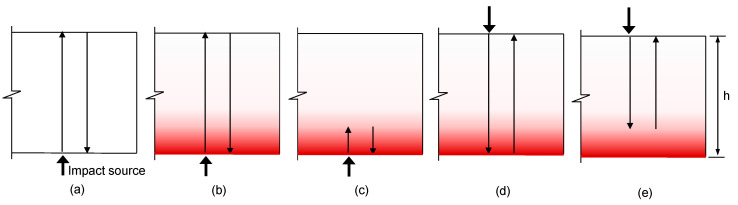
Several possible wave paths of non-propagating waves in solid and fire-damaged concrete slabs: (**a**,**b**,**d**) are the multiple reflections of P-waves between the two free surfaces on solid concrete slabs and the fire-damaged concrete slabs, caused by an impact source on either surface of the concrete slab specimen (the bottom surface for (**a**,**b**), and the top surface for (**d**)); (**c**,**e**) show the multiple reflections of P-waves between a free surface and an internal acoustic reflector at a high impedance mismatch, generated by an impact source on the bottom surface and top surface, respectively.

The dominant spectral responses (peak frequencies) of fire-damaged concrete, given by IE testing, were estimated from the reconstructed P-wave velocity profiles and temperature-dependent material properties described in the Appendix A of this article, using the discrete layered concrete model. It was assumed that fire-damaged concrete does not feature any fracture planes, such as cracks parallel to the concrete surface and/or surface-breaking cracks. Therefore, the spectral response of fire-damaged concrete, which was caused by applying an impact load on the free-surface of a concrete slab, is dominated by several non-propagating waves trapped in a plate (Figure 16a,b,d) or set up by the high acoustic impedance mismatch between severely damaged and mildly damaged concrete (Figure 16c,e).

The peak frequency of the thickness stretch mode in the fire-damaged concrete slab was approximated as follows:

(4) $$f_{IE,\;\mathrm{model}}=1/T_{Wave\;type}$$

where $T_{Wave\;type}$ is an apparent period of the thickness stretch mode (or S1 ZGV Lamb mode) and the subscript ‘Wave type’ indicates the type of thickness stretch mode in the fire-damaged concrete according to the wave path under different concrete conditions. The apparent period of the thickness stretch mode through the full thickness of the fire-damaged concrete (see Figure 16b), $T_{full\;thicknes}$, is expressed as follows:

(5) $$T_{full\;thickness}=2\overset{N}{\underset{i=1}{\sum }}\Delta h/\left[\beta_{i}\left(v_{i}\left(\theta \right)\right)C_{p,i}\left(\theta \right)\right]$$

where △*h* is the depth of each layer in the fire-damaged concrete model, *β_i_* is a correction factor [6] that depends on the temperature-dependent Poisson’s ratio of the concrete $v_{i}\left(\theta \right)$ [8], *C_p,i_* is the temperature-dependent P-wave velocity concrete, and the subscript *i* indicates the index of the concrete layer. 

**Figure 17:**
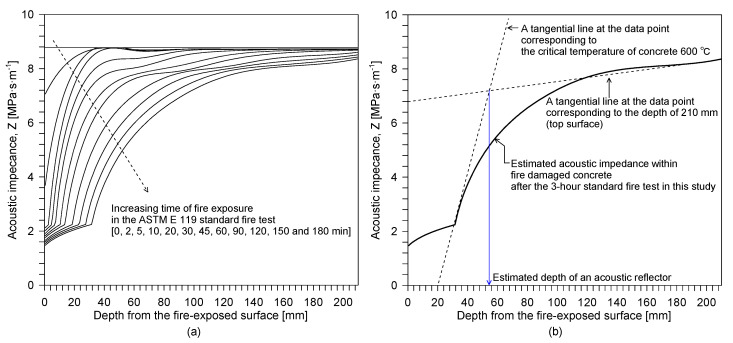
Acoustic impedance within fire-damaged concrete: (**a**) predicted acoustic impedance within fire-damaged concrete with increasing the time of fire exposure after the ASTM E 119 standard fire test, and (**b**) an example of determining the approximate depth of an acoustic reflector in fire-damaged concrete.

In contrast, Equations (5) and (6) are the approximate periods of the thickness stretch modes set up within the severely damaged concrete by an impact loading on the fire surface, $T_{severly\;damaged\;concrete}$, and within the mildly damaged concrete by an impact loading on the opposite surface to the fire-exposed surface, $T_{mild\;damaged\;concrete}$, respectively.

(6) $$T_{severly\;damaged\;concrete}=4\overset{N_{cr}}{\underset{i=1}{\sum }}\Delta h/\left[\beta_{i}\left(v_{i}\left(\theta \right)\right)C_{p,i}\left(\theta \right)\right]$$

(7) $$T_{mild\;damaged\;concrete}=2\overset{N}{\underset{i=N_{cr}}{\sum }}\Delta h/\left[\beta_{i}\left(v_{i}\left(\theta \right)\right)C_{p,i}\left(\theta \right)\right]$$

where $N_{cr}$ is the layer index corresponding to the critical depth of the high impedance mismatch (or the depth of an acoustic reflector) in fire-damaged concrete. Figure 17a shows the variation of acoustic impedance, *Z*, within fire-damaged concrete, which was determined by Equation (8),

(8) $$Z_{i}\left(\theta \right)=C_{p,i}\left(\theta \right)\rho_{p,i}\left(\theta \right)$$

where *ρ_i_* is the temperature-dependent mass density of concrete. It was difficult to determine the exact location of an acoustic reflector in the fire-damaged concrete because the acoustic impedance within fire-damaged concrete smoothly decreases at depths further from the fire-exposed surface; it does not show clear acoustic impedance mismatched layers. It can be inferred from the experimental study described in Section 3.1 that the true location of the acoustic reflector would be deeper than the approximated value of 20 mm, which was based on the constant P-wave velocity of 4100 m/s. Furthermore, it is reasonable to assume that the acoustic reflector is located deeper than the depth corresponding to the critical temperature of concrete, 600 °C. In this study, the approximate location of the high impedance mismatch was found at the intersection of the two tangential lines in severely damaged concrete (at the temperature corresponding to 600 °C), and at the top concrete surface (see Figure 17b). Note that the constant multiplier, 4, in Equation (6) is for incident waves reflected from the damaged concrete, with higher acoustic impedance, by the less damaged concrete with lower acoustic impedance [36].

**Figure 18:**
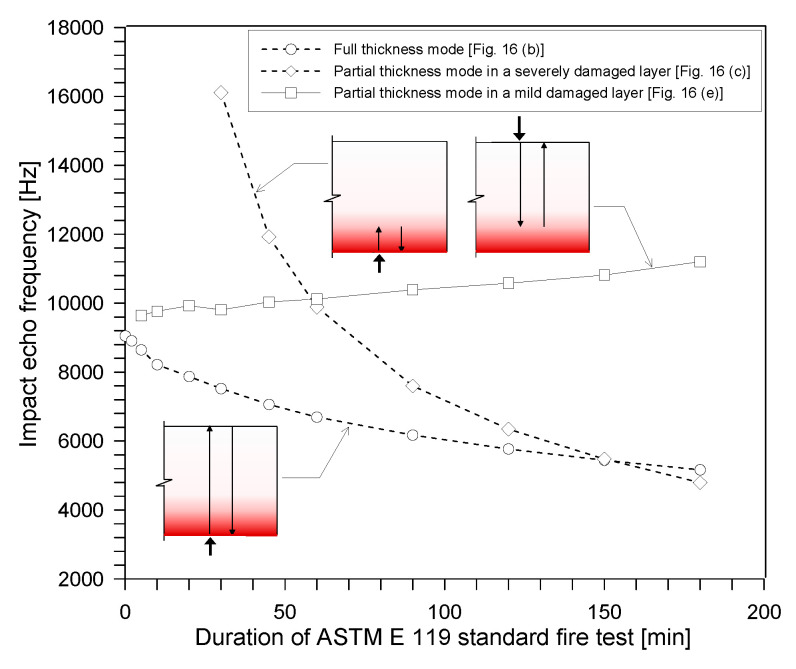
Variations in the thickness stretch mode frequency in the fire-damaged concrete slab specimens, estimated from the P-wave velocity profiles of fire-damaged concrete slabs, with different fire exposure times during the ASTM E 119 standard fire testing, reflecting full thickness, depth of severely damaged concrete and depth of mildly damaged concrete.

Figure 18 shows the variations in the thickness stretch mode frequency of the fire-damaged concrete slab specimens, which were estimated from the P-wave velocity profiles of fire-damaged concrete slabs with different fire exposure times during the ASTM E 119 standard fire testing. The initial P-wave velocity of sound concrete was obtained by incorporating the average IE frequency measured for sound concrete (i.e., 9170 Hz). The initial elastic modulus of concrete at room temperature was estimated to be 33.4 GPa using Equation (2), with initial values for the Poisson’s ratio and mass density of ν = 0.2 and ρ = 2230 kg/m^3^, respectively. The constant Poisson’s ratio value 0.2 is reasonable for the sound concrete [19,37]. The initial mass density value was the average of the measured mass densities of five sound concrete cylinders in this study. 

The predicted thickness stretch mode corresponding to the full thickness of the concrete slab specimen (210 mm) (see Figure 16b) is presented as open circles in Figure 18. The full thickness mode gradually decreases as the fire exposure time increases. Therefore, one possible reason for the frequency shift in the low frequency direction is the reduced stress wave velocities, due to the degradation of the material properties of concrete after being exposed to high heat (e.g., elastic modulus of concrete). After the 3-hour standard fire test, the estimated frequency was about 5100 Hz, which is comparable to the measured peak frequency of about 5165 Hz. 

The plots presented as open diamonds and open squares in Figure 18 show the variation of predicted IE frequency in concrete slabs, exposed to ASTM E 119 standard fire testing, measured on the bottom and top surfaces, respectively. At the bottom surface, the fire-exposed one, the frequency tends to decrease as the fire exposure time increases. After the 3-hour fire exposure, the predicted frequency for the bottom surface was about 5160 Hz, which showed a good agreement with the measured frequency of around 4700 Hz. From the top surface, the peak frequency tends to increase as the exposure time increases. This high frequency shift can be explained by the increase in depth of a new acoustic reflector (or the depth of severely damage concrete). It is similar to the frequency change due to a crack parallel to the concrete surface in concrete. After the 3-hour fire exposure, the predicted frequency for the top surface was about 10,343 Hz, which showed a good agreement with the measured frequency of around 10,200 Hz. 

## 4. Conclusions

In this study, a simplified model was developed to better interpret the IE data measured from fire-damaged concrete slabs. To show the validity of the model, a reinforced concrete slab specimen (100 mm (width) by 5000 mm (length) by 210 mm (thickness)) was fabricated in the laboratory. Heat damage in the concrete slab was induced by exposing the bottom surface of the concrete slab to the 3-h ASTM E 119 standard fire test. The dynamic responses of sound concrete and fire-damaged concrete were investigated by IE testing.

(1) The IE data showed that the dynamic response of the sound concrete slab was characterized by a single clear peak frequency of around 9300 Hz, which corresponds to the S1 zero group velocity Lamb mode frequency (i.e., thickness stretch mode) from the IE test. It was observed that the dynamic response of the fire-damaged concrete slab was dominated by several non-propagating waves, which results in two groups of dominant peak frequencies: low-frequency shift around 4000 Hz to 5100 Hz, and high-frequency shift around 10,200 Hz.
(2) The P-wave velocity profile reconstructed by the discrete layered concrete model indicates that the frequency shifts in the fire-damaged concrete are caused by the reduced P-wave velocity, and the multiple reflections of elastic waves due to the abrupt change in the P-wave velocity gradient near the fire-exposed surface.
(3) The validity of the discrete layered concrete model was verified by comparing the P-wave velocity profiles from the model to those measured by ultrasonic pulse velocity measurements of the five core samples extracted from the fire-damaged concrete.
(4) The results in this study could be useful in the better interpretation of the dynamic responses of fire-damaged concrete slabs determined by IE testing, and for the condition assessment of fire-damaged concrete.
(5) The results in this study were obtained from a fabricated concrete slab specimen in the laboratory. More studies are still needed to obtain more general conclusions regarding the feasibility of using the discrete fire-damaged model when evaluating fire-damaged concrete via IE testing.

## Figures and Tables

**Table 1:** Summary of a statistical analysis: K-S test results and mean, standard deviation and coefficient of variation of data sets from two sound and fire-damaged concrete slabs in this study.

ID	K-S Test Results		Mean [Hz]	Standard Deviation [Hz]	COV [%]
	h	*p*-Value			
Group 1	0	0.4415	9117	189	2.08
Group 2	0	0.0683	5034	792	15.74

Note: Group 1: sound concrete, Group 2: fire-damaged concrete after 3-hour ASTM E 119 standard fire test; h is the result of the K-S test. h = 0 indicates does not reject the null hypothesis (normal distribution) at the significant level of 5% in this study.

**Figure A1:**
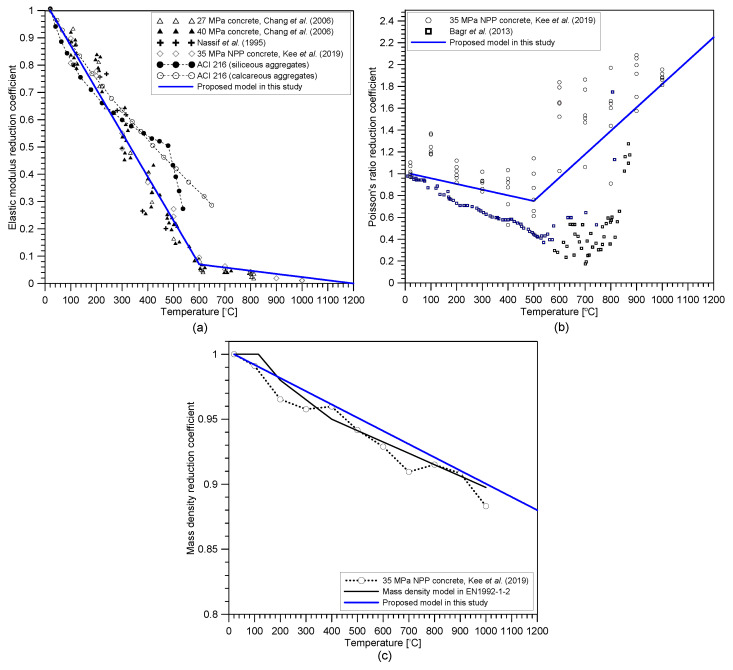
Variations of calibration factors $\Phi_{E},\Phi_{\nu }\;$ and $\Phi_{\rho }$ that describe the temperature-dependent natures of the three concrete properties: dynamic elastic modulus, $E_{d\;}$, dynamic Poisson’s ratio, $\nu_{d}$, and mass density, ρ.

## Appendix A. Temperature-Dependent Concrete Properties

In this study, the temperature-dependent properties of concrete that determine the dynamic response of concrete (dynamic elastic modulus, $E_{d\;}\left(\theta \right)$, dynamic Poisson’s ratio, $\nu_{d\;}\left(\theta \right)$, and mass density, ρ(θ)) are expressed as the reference values of room temperature,$\theta_{R}\;\left(=20\;℃\right),$ for each parameter multiplied by calibration factors $(\Phi_{E_{d}},\;\Phi_{\nu_{d}}$ and $\Phi_{\rho_{d}})$, which describes the temperature-dependent variation of each parameter, as follows:

(A1) $$E_{d\;}\left(\theta \right)=\;\Phi_{E_{d}}\;E_{d\;}\left(\theta_{R}\right),$$

(A2) $$\nu_{d\;}\left(\theta \right)=\;\Phi_{\nu_{d}}\;\nu_{d\;}\left(\theta_{R}\right),$$

and

(A3) $$\rho \left(\theta \right)=\;\Phi_{\rho }\;\rho \left(\theta_{R}\right).$$

Figure A1a–c show the variations in $\Phi_{E_{d}},\;\Phi_{\nu_{d}}\;$ and $\Phi_{\rho }$ presented as open circles in the figures that were obtained from an experimental study based on concrete cylinders made with the same mixing proportions as those used for the fabrication of the concrete slab specimen in this study [19]. The three critical parameters ($E_{d\;}$, $\nu_{d\;}$, and ρ) tend to decrease as the maximum temperature of concrete increases. Among them, exposure to high temperature was the most influential for $E_{d\;}$, as the $E_{d}$ values linearly decreased as the temperature increased from 20 ℃ to 600 ℃. At 600 ℃, the $E_{d}$ values were only about 7% of the initial values at room temperature. After 600 ℃, the $E_{d\;}$ values further decreased with temperature, with a slower reduction rate, and dropped to almost zero at 1000 ℃. The abrupt reduction of the $E_{d}$ value of concrete can be explained by the damage of the concrete at the microstructure level, i.e., the decomposition of cement paste and the expansion of aggregates in concrete. After the temperature of concrete increases to between 400 ℃ and 550 ℃, the degradation of calcium hydroxide (Ca(OH)_2_ → CaO + H_2_O) causes the shrinkage of the concrete and changes in the microstructure of the cement pastes. At temperatures between 500 ℃ and 600 ℃, the phase of quartz (SiO_2_) in aggregates converts from α to β, which causes a volume expansion of about 0.45%, and promotes microcracking upon increasing the internal stress of concrete, especially for siliceous aggregate concrete.

In this study, approximate equations representing the temperature-dependent behaviors of the three concrete parameters ($\Phi_{E_{d}},\;\Phi_{\nu_{d}}$ and $\Phi_{\rho }$) were established using regression analyses based on bilinear equations, as follows:

(A4) $$\{\begin{array}{c} \frac{\Phi_{i}\;-\alpha }{1-\alpha }+\frac{\theta -\theta_{R}}{\theta_{A}-\theta_{R}}=1\;\;\;\;\;\;\;\;\;\;\;\;\;\;\;\;\;\;\;\;\;\;for\;\theta_{R}\leq \theta \leq \theta_{A}\\ \frac{\Phi_{i}\;-\alpha }{\beta -\alpha }+\frac{\theta -\theta_{A}}{\theta_{B}-\theta_{A}}=1\;\;\;\;\;\;\;\;\;\;\;\;\;\;\;\;\;\;\;\;\;\;\;\;for\;\theta_{A}<\theta \leq \theta_{B} \end{array}$$

where $\Phi_{i}$ is the temperature-dependent variation of a parameter i ($E_{d}$,$\;\nu_{d}$ or ρ)$,\;\theta_{A}$ is the first critical temperature of concrete (=600 ℃) corresponding to the inflection points of each parameter, $\theta_{B}$ is the second critical temperature of concrete beyond which concrete completely lose its mechanical properties (i.e., the elastic modulus of concrete becomes zero), and the α and β values are the $\Phi_{i}$ at the two critical temperatures of concrete (600 ℃ and 1200 ℃, respectively). Table A1 summarizes the values of the numerical parameters that determine the approximate equations for the three temperature-dependent properties ($E_{d}$,$\;\nu_{d}$ or ρ). The three approximate equations are presented as blue lines in Figure A1a–c, respectively.

**Table A1:** Estimated IE peak frequencies using the simplified fire damage model.

	$\theta_{R}$	$\theta_{A}$	$\theta_{B}$	α	β
$\Phi_{E}$	20	600	1200	0.07	0
$\Phi_{\nu }$	20	500	1200	0.75	2.5
$\Phi_{\rho }$	20	1200	-	0.90	-
